# CtIP Regulates Mitotic Spindle Assembly by Modulating the TPX2-Aurora A Signaling Axis

**DOI:** 10.3390/cells11182814

**Published:** 2022-09-08

**Authors:** Wonkyung Oh, Ting Ting Wu, Seo-Yeon Jeong, Ho Jin You, Jung-Hee Lee

**Affiliations:** 1 Laboratory of Genomic Instability and Cancer Therapeutics, Cancer Mutation Research Center, School of Medicine, Chosun University, 375 Seosuk-dong, Gwangju 61452, Korea; 2 Department of Pharmacology, School of Medicine, Chosun University, 375 Seosuk-dong, Gwangju 61452, Korea; 3 Department of Cellular and Molecular Medicine, School of Medicine, Chosun University, 375 Seosuk-dong, Gwangju 61452, Korea

**Keywords:** CtIP, spindle, mitosis, TPX2, kinetochore, spindle assembly checkpoint

## Abstract

CtBP-interacting protein (CtIP) plays a critical role in controlling the homologous recombination-mediated DNA double-stranded break (DSB) repair pathway through DNA end resection, and recent studies suggest that it also plays a role in mitosis. However, the mechanism by which CtIP contributes to mitosis regulation remains elusive. Here, we show that depletion of CtIP leads to a delay in anaphase progression resulting in misaligned chromosomes, an aberrant number of centrosomes, and defects in chromosome segregation. Additionally, we demonstrate that CtIP binds and colocalizes with Targeting protein for Xklp2 (TPX2) during mitosis to regulate the recruitment of TPX2 to the spindle poles. Furthermore, depletion of CtIP resulted in both a lower concentration of Aurora A, its downstream target, and very low microtubule intensity at the spindle poles, suggesting an important role for the CtIP-TPX2-Auroa A complex in microtubule dynamics at the centrosomal spindles. Our findings reveal a novel function of CtIP in regulating spindle dynamics through interactions with TPX2 and indicate that CtIP is involved in the proper execution of the mitotic program, where deregulation may lead to chromosomal instability.

## 1. Introduction

CtBP-interacting protein (CtIP), also known as RBBP8, is a protein that is evolutionarily conserved from yeast to humans. Inactivation of both CtIP alleles causes early embryonic lethality in mice [1,2] and mice with haploid insufficiency are viable but displays genomic instability and tumorigenesis [2,3]. CtIP is an 897 amino acid protein consisting of an *N*-terminal dimerization domain, two coiled-coil domains, and specific motifs that bind interacting proteins. It was originally identified as a cofactor of the transcriptional repressor CtBP, and it regulates the expression of the transcription factors LMO4, Ikaros, E2F, and TFIIB [4,5,6,7].

CtIP is known to play an important role in DNA end resection, a process that in mammalian cells is triggered by the presence of damaged double stranded DNA and is followed by homologous recombination (HR) repair [8]. The association of CtIP with the MRN complex (Mre11-Rad51-Nbs1) is a critical step in initiating DNA end resection [9], occurring after CtIP has been phosphorylated in a CDK1-mediated process when it binds to both the MRN complex and BRCA1 [10]. BRCA1 then regulates the activation of both the G2/M checkpoint and Chk1 via ATM/ATR during the DNA damage response. Interactions between CtIP and BRCA1 are important for the ubiquitination and chromatin association of CtIP [11]. Therefore, the association of CtIP with diverse cancer-related proteins leads to multifunctional roles in cell signaling, including DNA damage checkpoint, DNA repair, cell survival, and cell cycle control [3].

The mitotic spindle is essential for cell division and is responsible for the accurate segregation of chromosomes into to the two daughter cells during mitosis, which is critical for maintaining genome integrity [12,13,14]. The mitotic spindle is a bipolar structure consisting of microtubules (MTs), dynamic polar structures polymerized from α/β tubulin heterodimers, and microtubule associated proteins (MAPs). Spindle assembly is initiated by MT nucleation and generated from MT organizing centers (MTOCs) at the centrosome, where polarized growth of MTs begins at the centrosome with minus ends tethered to the spindle poles and plus ends extending outward [15,16,17]. Targeting protein for XKlp2 (TPX2) has been identified as an MT-associated protein and is one of the MAPs that are essential for mitotic spindle assembly. TPX2 localizes to the nucleus during interphase and is recruited to the mitotic spindles during mitosis [18,19]. TPX2 binds and activates Aurora A, a kinase that is subsequently targeted to the spindle poles [20,21,22]. As a part of the Hepatoma up-regulated protein (HURP) complex, TPX2 also mediates Ran-dependent bipolar spindle formation [23,24,25]. Moreover, TPX2 associates with other substrates, including Eg5, TACC3, and Adducin-1 to play a central role in regulating spindle assembly, centrosome separation, and spindle pole integrity [26,27,28]. 

In the present study, we show that CtIP has mitosis-specific functions. CtIP depletion leads to a delay in mitosis, with multipolar spindles and misaligned chromosomes. We provide evidence that CtIP binds to TPX2 and regulates the accumulation of TPX2 on kinetochores and that phenotypes resulting from the downregulation of CtIP resemble those observed when TPX2 is impaired. Furthermore, we show that a CtIP knockdown affects the efficient localization of Aurora A, a downstream target of TPX2, to the spindle poles. Our results revealed a previously unknown role of CtIP in the direct regulation of microtubule dynamics at the centrosomal spindles via interaction with TPX2. 

## 2. Materials and Methods

### 2.1. Cell Lines and Transfection 

The human cervix adenocarcinoma HeLa cells, human osteosarcoma U2OS cells, and human embryonic kidney HEK293T cells were obtained from American Type Culture Collection (ATCC, Rockville, MD, USA) and cultured at 37 °C, 10% CO_2_ in Dulbecco’s Modified Eagle’s Medium (DMEM) with high glucose plus 10% fetal bovine serum (FBS), penicillin, and streptomycin. Plasmids were introduced into cells by transfection using TurboFect (Thermo Fisher Scientific laboratories, Middlesex, MA, USA) according to the manufacturer’s instructions. siRNAs were transfected at a final concentration of 50 nM using RNAiMax (Invitrogen, Carlsbad, CA, USA).

### 2.2. Plasmids

Hemagglutinin (HA)-tagged TPX2 expression vector and pmCherry/TPX2 were purchased from Addgene (Addgene, Watertown, MA, USA). TPX2 was amplified by PCR, and the PCR products were subcloned into pcDNA3-HA vector. PCR primer sequences were forward, 5′-ataagaatgcggccgcggatgtcacaagttaaa-3′; reverse, 5′-acgcgtcgacttagcagtggaatcg-3′. Full length human wild-type CtIP was obtained by PCR from human HeLa cDNA and cloned into pcDNA3.1-Flag vector. PCR primer sequences were forward, 5′-CGGATATCCGATGAACATCTTGGGA-3′; reverse, ACGCGTCGACCTATGTCTTCTGCTC-3′. pEGFP/H2B vector was obtained from Addgene. The all constructs were confirmed by automated DNA sequencing. 

### 2.3. RNA Interference and Transfection

The three siRNA oligonucleotides were used to knockdown CtIP included siCtIP-1: 5′-GCUAAAACAGGAACGAATCdTdT-3′ [29], siCtIP-2: 5′-GGACCUUUGGACAAAACUAdTdT-3′ [30], siCtIP-3 (3′-UTR): 5′-GAAGGAUGAAGGACAGUUUdTdT-3′ and TPX2 siRNA sequence is 5′-GAACAATCCATTCCGTCAAATdTdT-3′. Scrambled siRNA was used as a negative control (Bioneer, Daejeon, Korea). The control or CtIP siRNA were transiently transfected into the cells using Lipofectamine RNAiMAX (Invitrogen) according to the manufacturer’s instructions. After 48 h, the knockdown of CtIP was judged by Western blot analysis. 

### 2.4. Antibodies

The following antibodies were used for immunostaining; Rabbit anti-CtIP (D76F7, #9201, cell signaling, Danvers, MA, USA), mouse anti-CtIP (D-4, 271339, Santa Cruz Biotechnology, Dallas, TX, USA), rabbit anti-TPX2 (NB500-179, NOVUS, Centennial, CO, USA), rabbit anti-γ tubulin (T3559, Sigma, St Louis, MO, USA), mouse anti-α-tubulin (#14-4502-82, Invitrogen), rabbit anti-human ANA-Centromere CREST (90C-CS1058, Fitzgerald, Wicklow, Ireland), mouse anti-BubR1 (ab4637, abcam, Cambridge, UK), rabbit anti-Mad1 (sc-67338, Santa Cruz Biotechnology), rabbit anti-Mad2 (A300-300A, Bethly, Fortis life sciences, Waltham, MA, USA), rabbit anti-phospho Aurora A T288 (3079, Cell Signaling, Danvers, MA, USA), mouse anti-RCC1 (F-2, Santa Cruz Biotechnology). The following antibodies were used for Western blot analysis; mouse anti-CtIP (D-4, 271339, Santa Cruz Biotechnology), rabbit anti-TPX2 (NB500-179, NOVUS), rabbit anti-CyclinB1 (H-433, Santa Cruz Biotechnology), rabbit anti-Histone H3 (06-755, Millipore, Burlington, MA, USA), rabbit anti-phospho-Histone H3 Ser10 (9701, Cell Signaling), mouse anti-β-actin (sc-47778, Santa Cruz Biotechnology), mouse anti-Flag (M2, Sigma) and mouse anti-HA (F-7, Santa Cruz Biotechnology). For Immunoprecipitation assay, mouse anti-Flag (M2, Sigma) and rabbit anti-HA (600-401-384, Rockland, Limerick, PA, USA) were used. 

### 2.5. Immunofluorescence Microscopy and Live-Cell Analysis

HeLa cells grown on sterile coverslips were transfected with plasmids or siRNAs as described above. The cells were fixed with 4% paraformaldehyde for 15 min. In some cases, 5 min pre-extraction procedures with PHEM (60 mM PIPES, 25 mM HEPES, 2 mM MgCl_2_, 10 mM EGTA plus 0.5% Triton X-100) were performed before fixation. The cells were then permeabilized with 0.5% Triton X-100 for 5 min and blocked with 5% BSA for 30 min at room temperature before incubating with the relevant primary antibodies diluted in PBS plus 2.5% BSA for 2 h. After rinsing in PBST (0.025% tween 20 in phosphate-buffered saline), the cells were incubated with Alexa-fluorescence conjugated secondary antibodies for 1 h at room temperature. The secondary antibodies were anti-rabbit Alexa Fluor-488 or anti-mouse Alexa Fluor-488, anti-mouse Alexa Fluor-488 or anti-rabbit Alexa Fluor-594, and anti-human Alexa Fluor594 (Molecular Probes, Eugene, OR, USA). The coverslips were mounted on glass slides with fluoroshield containing 4′,6-diamidino-2-phenylindole (DAPI, Vector Laboratories, Burlingame, CA, USA). Fluorescence staining was visualized, and images were collected a confocal microscope (Zeiss LSM 510 Meta; Carl Zeiss, Jena, Germany). For cold stable microtubule assays, media from cultured cells was removed and replaced with ice-cold DMEM. Cells were then incubated on ice for 10 min and immunofluorescence staining was performed as described above. For microtubule regrowth assays, siRNA-transfected cells were incubated on ice for 1 h and rewarmed at 37 °C for 45 s and 90 s before fixation. Immunostaining was carried out as described above. For live-cell imaging analysis, cells were transfected with GFP-tagged histone H2B-pcDNA3 followed by time lapse microscopy using an Axio Observer microscope (Carl Zeiss) maintained at 37 °C in 5% CO_2_. Images were acquired at 3–5 min intervals for 12 h and analyzed using Zen 3,4 blue edition software (Carl Zeiss).

### 2.6. Immunoprecipitation and Western Blot Analyses

For immunoprecipitation assays, cells were transfected with plasmids or arrested with nocodazole to collect mitotic cells. Cells were lysed in lysis buffer (20 mM Tris-HCl [pH 8.0], 0.1% SDS, 0.1% NP-40, 50 mM NaCl, 1 mM EDTA, 20 mM β-glycerophosphate, 1 mM Na_3_-vanadate, 50 mM NaF, 5% glycerol, 10 μg/mL leupeptin, 10 μg/mL pepstatin A, 10 μg/mL aprotinin, 1 mM PMSF and 1 mM DTT). Extracts were centrifuged (16,000× *g*, 10 min at 4 °C) and then pre-cleared with protein G sepharose and IgG for 1 h. Primary antibodies were added to the precleared lysates and incubated for 2 h at 4 °C. Following a 2 h incubation with protein G sepharose beads at 4 °C, the beads were washed three times with TNET lysis buffer, and the bound protein complex was resolved in SDS sample buffer. For Western blotting, the transfected or treated cells were harvested and lysed in lysis buffer (50 mM Tris-HCl [pH 8.0], 0.1% SDS, 1.0% NP-40, 50 mM NaCl, 1 mM EDTA, 0.5% sodium deoxycholate, 80 mM β-glycerophosphate, 1 mM Na_3_-vanadate, 50 mM NaF) supplemented with protease inhibitors, 1 mM PMSF, and 1 mM DTT. The collected cells were sonicated and cleared by centrifugation. The lysates were boiled for 5 min, and proteins were separated by SDS-PAGE and transferred to PVDF. The membrane was blotted with the indicated antibodies overnight, and the signal was detected using chemiluminescence.

### 2.7. Cell Cycle Synchronization

To synchronize in the G1/S-phase boundary, a double thymidine block was accomplished by the growing cell in a medium containing 2 mM thymidine (Sigma-Aldrich, St Louis, MO, USA) for 18 h, washing with PBS two times, incubating in a medium without thymidine for 9 h. It was then reincubated in medium containing 2 mM thymidine for an additional 17 h. After the second block, cells were washed twice with PBS and given a fresh medium until processed for analysis. For mitotic cell synchronization, cells were treated with 100 ng/mL nocodazole for 16 h.

### 2.8. Statistical Analysis

Statistical comparisons were carried out using the two-tailed paired Student′s *t*-test and Mann–Whitney test. Results with a value of *p* < 0.05 (*) and *p* < 0.01 (**) were considered statically significant; ns = nonsignificant. Analyses were carried out with GraphPad Prism (GraphPad software, San Diego, CA, USA) and Microsoft Excel (Microsoft, Redmond, WA, USA). All data were represented as mean ± SD, and all experiments were performed at least three times unless otherwise stated.

## 3. Results

### 3.1. CtIP Is a Mitotic Spindle Associated Protein

**Figure 1:**
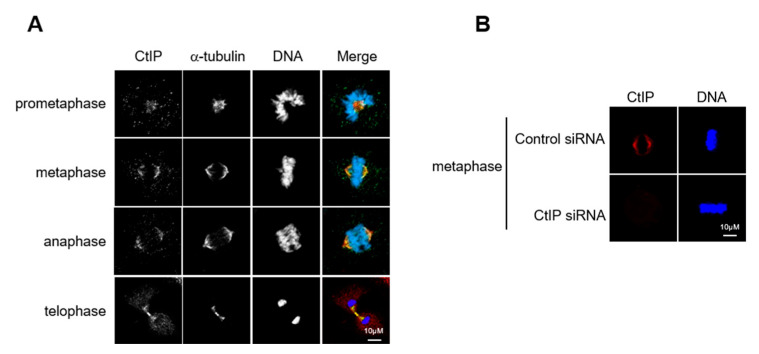
CtIP is associated with spindles during mitosis. (**A**) HeLa cells undergoing mitosis were fixed and stained with anti-CtIP and anti-α-tubulin antibodies. DNA was visualized using DAPI. The images were captured using a confocal microscope. (**B**) HeLa cells transfected with control siRNA, and CtIP siRNA undergoing mitosis were fixed and stained with anti-CtIP antibody. DNA was visualized using DAPI. The images were captured using a confocal microscope.

A previous study suggested that CtIP contributes to proper chromosome alignment by interacting with the MRN complex [31]. To better understand the role of CtIP in mitosis, we examined CtIP localization during mitotic progression in HeLa cells and observed that CtIP was localized to the mitotic spindle throughout this process (Figure 1A,B). Staining for CtIP suggested that it was spread across the entire spindle during prometaphase and metaphase but was absent from the pole during metaphase. At telophase, CtIP staining became more diffuse throughout the cytosol and appeared in the reforming nucleus. These results suggest that CtIP is a spindle-associated protein throughout mitosis and potentially plays a previously unrecognized role as an interacting partner in mitotic spindle function.

### 3.2. Knockdown of CtIP Results in Mitotic Delay 

**Figure 2:**
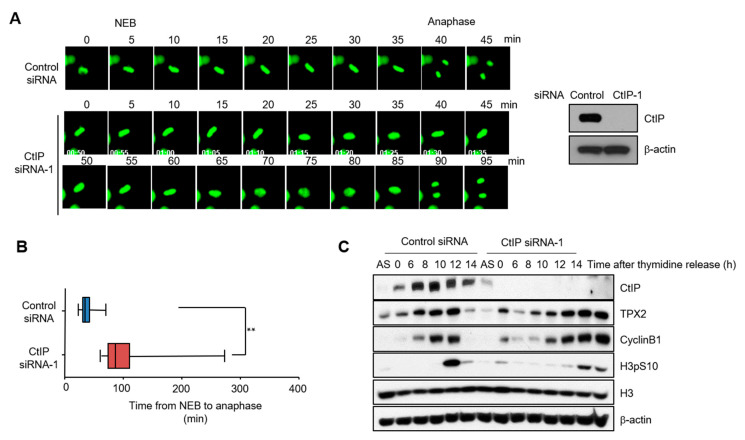
CtIP depletion causes improper progression of mitosis. (**A**) The progression of mitosis in HeLa cells was monitored by time-lapse microscopy. HeLa cells were transfected with control or CtIP-1 or siRNA. After 48 h, control and CtIP-depleted HeLa cells were seeded in 12-well plates and transfected with GFP-tagged histone H2B. Fluorescent images were obtained every 5 min starting at the stage of nuclear envelope breakdown. (**B**) A quantification of the time from nuclear envelope breakdown to anaphase onset in control cells and CtIP-depleted cells. Bars represent the mean ± SD from three independent experiments. **, *p* < 0.01, compared to control cells. (**C**) Delayed mitosis progression in CtIP-depleted cells was confirmed by prolonged phosphorylation of histone H3 (pH3S10). Control and CtIP-depleted HeLa cells were synchronized with a double thymidine block to arrest at the G1/S boundary and released from this block for indicated times. Total proteins collected at the indicated times after release were analyzed by Western blotting using anti-pH3S10 antibody. Histone H3 antibody was used as a loading control.

We next addressed the functional significance of CtIP localization to the mitotic spindles. Mitotic progression was monitored using time-lapse imaging of histone H2B in HeLa cells that has been transfected with an siRNA to inhibit CtIP expression. Immunoblotting confirmed that CtIP expression was ≥80% lower in HeLa cells transfected with CtIP siRNA than in those transfected with control siRNA (Figure 2A, right panel). We then transfected GFP-tagged H2B into control and CtIP-depleted HeLa cells and synchronized the cell cycle by release from a double thymidine block. Control cells (*n* = 65) started to enter mitosis 7 h after release from the double thymidine block, and the time from nuclear envelope breakdown to complete anaphase was ~40 min. CtIP-knockdown cells entered mitosis with similar kinetics but showed a wide variability in mitosis duration, which lasted up to ~90 min (Figure 2A,B). To rule out off-target effects of the CtIP siRNA, we tested the kinetics of anaphase onset with a different siRNA (CtIP siRNA-2) and observed the same delay of anaphase onset (Figure S1A–C). CtIP siRNA-1 was used in most of the CtIP knockdown experiments in this study, unless otherwise indicated. A similar phenotypic change of mitotic progression was observed upon knockdown of CtIP in U2OS cells (Figure S1D,E). When CtIP-depleted cells were complemented with siRNA-resistance wild-type hemagglutinin (HA)-tagged CtIP, they were rescued from the improper progression of mitosis (Figure S1F,G). Immunoblot analysis suggested prolonged phosphorylation of histone H3 at serine 10, a marker of mitotic cells, in CtIP-depleted cells (Figure 2C), further supporting an apparent mitotic delay in synchronized CtIP-depleted cells. 

### 3.3. CtIP Depletion Leads to Chromosome Misalignment and Improper Spindle Formation during Mitosis

**Figure 3:**
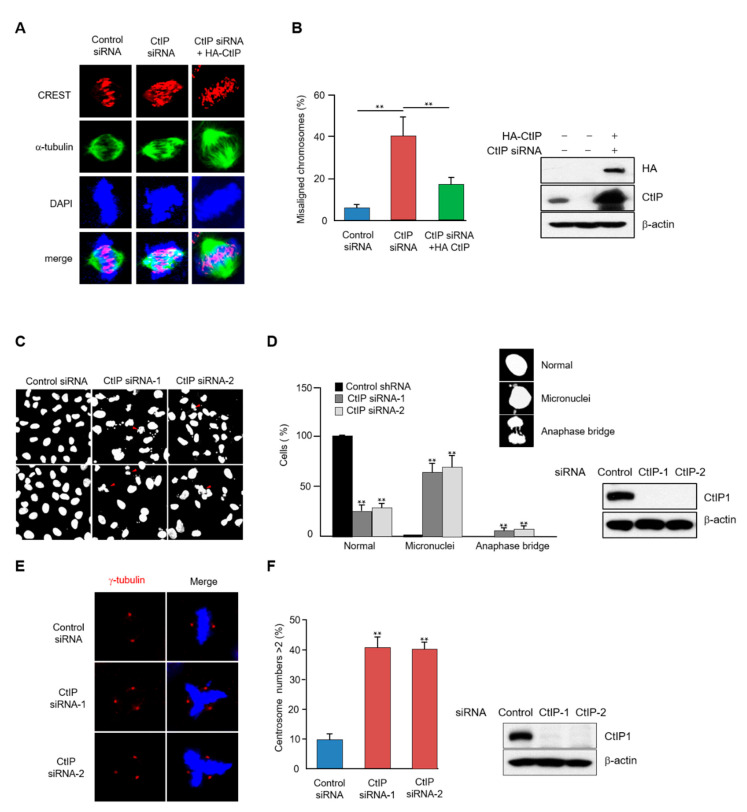
Depletion of CtIP leads to impaired chromosome alignment and improper spindle formation during mitosis. (**A**) The alignment of chromosomes in metaphase was observed for control HeLa cells, CtIP-depleted HeLa cells, and CtIP depleted HeLa cells reconstituted with HA-CtIP immunofluorescence microscopy with α-tubulin antibody for microtubules (green), CREST antibody for kinetochores (red) and DAPI staining for chromosomes. (**B**) A quantification of cells (*n* = 100) with misaligned chromosomes in metaphase. The results represent the mean ± SD from three independents experiments. **, *p* < 0.01, compared to control siRNA-transfected cells. (**C**) Images of mitotic defects in control and CtIP-depleted HeLa cells. Arrows indicate micronuclei and anaphase bridges. (**D**) Quantification of aberrant nuclear morphology in control and CtIP-depleted HeLa cells (*n* = at least 100 cells); **, *p* < 0.01. (**E**) Control and CtIP-depleted HeLa cells were fixed and stained with anti-γ-tubulin antibody and DNA was visualized using DAPI staining. (**F**) A quantification of cells (*n* = 100) with centrosomal amplifications. The results represent the mean ± SD from three independents experiments. **, *p* < 0.01, compared to control cells. To confirm depletion of CtIP, whole cells lysates were analyzed by Western blotting with anti-CtIP and anti-β-Actin antibodies.

Abnormal mitotic progression may cause aberrant spindle formation and inappropriate chromosome segregation [32]. We thus examined the effects of CtIP depletion on each of these two phenotypes. The organization of mitotic spindles in CtIP knockdown cells was visualized using an α-tubulin antibody and 4′,6-diamidino-2-phenylindole (DAPI) staining. The abnormal spindle structures were showed in the metaphase spindle of the CtIP-depleted cells in the presence of MG132 to arrest at metaphase. Notably, overexpression of wild-type HA-tagged CtIP restored proper spindle morphology and chromosome alignment in CtIP-depleted cells (Figure 3A,B).

Next, a morphological examination of the nucleus was carried out, revealing that CtIP depletion markedly increased the number of micronucleated cells compared to the control cell number (Figure 3C,D). Moreover, reconstituting these cells with wild-type HA-tagged CtIP restored the number of micronucleated cells (Figure S2A,B). This result suggests that defects occurred during chromosome segregation, and indeed, a significant number of chromatin bridges were detected in the CtIP-depleted cells (Figure 3C,D). To provide further insight into the abnormal spindle phenotype, we examined the integrity of centrosomes by staining with the centrosomal marker γ-tubulin and observed that ~ 40% of the CtIP-depleted cells displayed more than two centrosomes per mononucleated cells as compared to 5% for the control cells (Figure 3E,F). Taken together, our data indicate that CtIP contributes to proper chromosome alignment and spindle formation during mitosis.

### 3.4. Depletion of CtIP Induces Activation of Spindle Assembly Checkpoint

**Figure 4:**
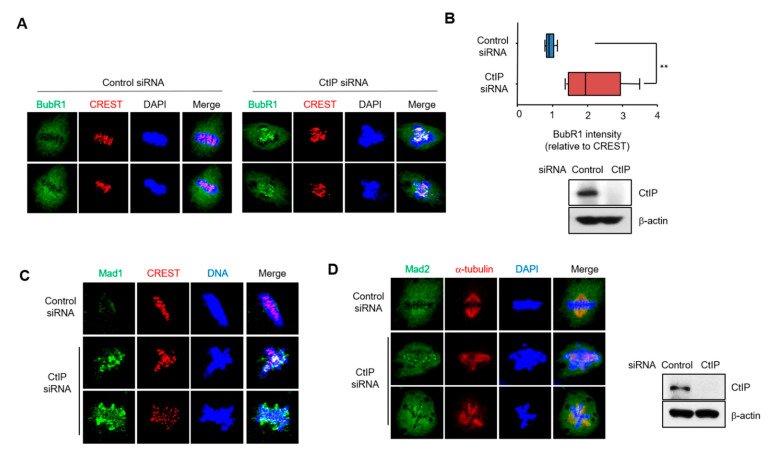
Loss of CtIP induced activation of spindle assembly checkpoints. (**A**) Kinetochore localization of the spindle assembly checkpoint regulator, BubR1, was visualized in control and CtIP-depleted HeLa cells using immunofluorescence staining. BubR1 is shown in green, and CREST, which localizes to kinetochores, is red. DNA was visualized using DAPI. (**B**) Quantification of the fluorescence intensity of BubR1normalized to that of CREST. Graphs represent the mean ± SD from three independents experiments. **, *p* < 0.01, compared to control cells. (**C**,**D**) Mad1 and CREST (**C**) and Mad2 and α-tubulin (**D**) were visualized in control and CtIP-depleted HeLa cells using immunofluorescence staining. DNA was stained with DAPI. CtIP-depletion was confirmed by Western blotting with anti-CtIP and anti-β-Actin antibodies.

In normal cells, an inaccurate microtubule-kinetochore attachment will triggers the activation of a spindle assembly checkpoint (SAC, also known as mitotic checkpoint) and induces mitotic arrest by delaying sister chromatid separation until all chromosomes are properly attached to the spindle [33]. SAC defects are associated with chromosomal instability, aneuploidy, and cancer predisposition. The proteins responsible for the SAC include the Mad (mitotic-arrest deficient) proteins Mad1, Mad2, and Mad3 (BubR1 in humans) and the Bud (budding uninhibited by benzimidazole) proteins Bud1 and Bud3 [16]. We examined whether depletion of CtIP affected the localization of SAC proteins at kinetochores. As shown by immunofluorescence microscopy, the signal intensity of BubR1 at the kinetochores was increased in the CtIP-depleted cells compared to that in the control cells, and quantification of the BubR1-stained cells showed a 2-fold increase in fluorescence intensity in the CtIP-knockdown cells (Figure 4A,B). In addition, the levels of Mad1 and Mad2 at the kinetochores appeared to be higher in the CtIP-knockdown cells (Figure 4C,D). These data suggested that CtIP depletion causes persistent SAC activation due to mitotic spindle assembly defects.

### 3.5. CtIP Interacts and Colocalizes with TPX2 

**Figure 5:**
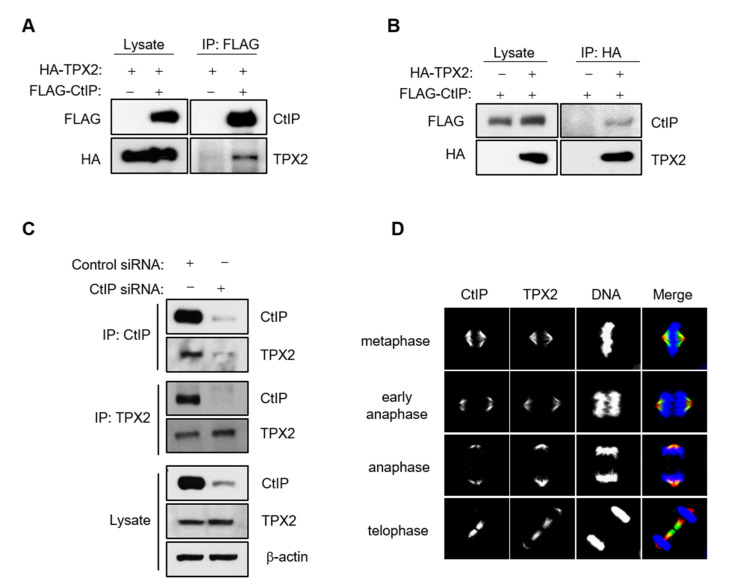
CtIP interacts with TPX2. (**A**,**B**) CtIP coimmunoprecipitates with TPX2. Total cell lysates (1 mg) from HEK293T cells transfected with Flag-tagged full length CtIP and HA-tagged full length TPX2 were immunoprecipitated with anti-Flag (**A**) or anti-HA (**B**) antibodies. Immunoblotting was then performed with the indicated antibodies. (**C**) Total cell lysates from HEK293T cells transfected with control siRNA and CtIP siRNA were immunoprecipitated with anti-CtIP or anti-TPX2 antibodies. Immunoblotting was then performed with the indicated antibodies. (**D**) The cellular localization of CtIP and TPX2 during mitosis was monitored using Immunofluorescence microcopy. Asynchronous HeLa cells were fixed and stained with anti-CtIP and anti-TPX2 antibodies. Representative images show that CtIP colocalizes with TPX2 at the kinetochore from prometaphase through anaphase.

TPX2, which is required for mitotic spindle formation and function [19], was identified as a putative CtIP-binding protein by quantitative BAC-GFP interactomics (QUBIC) analysis [34]. To explore this further, interactions between CtIP and TPX2 were examined by transient co-transfection of HEK293T cells with HA-tagged TPX2 and Flag-tagged CtIP followed by immunoprecipitation and Western blotting. Cell lysates were immunoprecipitated with an anti-Flag antibody, and the immunoprecipitates were probed with and anti-HA antibody, indicating that Flag-CtIP was bound to HA-TPX2 (Figure 5A). In reciprocal experiments, HA-TPX2 also coimmunoprecipitated with Flag-CtIP (Figure 5B). We further analyzed the interaction between endogenous CtIP and TPX2. We found that that endogenous CtIP and TPX2 could be reciprocally co-immunoprecipitated in control cells but not CtIP-depleted cells (Figure 5C). We then examined the colocalization of CtIP and TPX2 during mitosis. Briefly, HeLa cells were synchronized at the G1/S boundary using a double thymidine block, released into the mitotic cell cycle, and subjected to immunofluorescence analysis with anti-CtIP and anti-TPX2 antibodies to monitor colocalization throughout the entire cell cycle. The results indicated that CtIP extensively colocalized with TPX2, particularly on kinetochores during metaphase, anaphase, and telophase (Figure 5D). Taken together, these results suggest that CtIP regulates TPX2 function throughout mitosis. 

### 3.6. CtIP Depletion Leads to Impaired TPX2 Signaling

**Figure 6:**
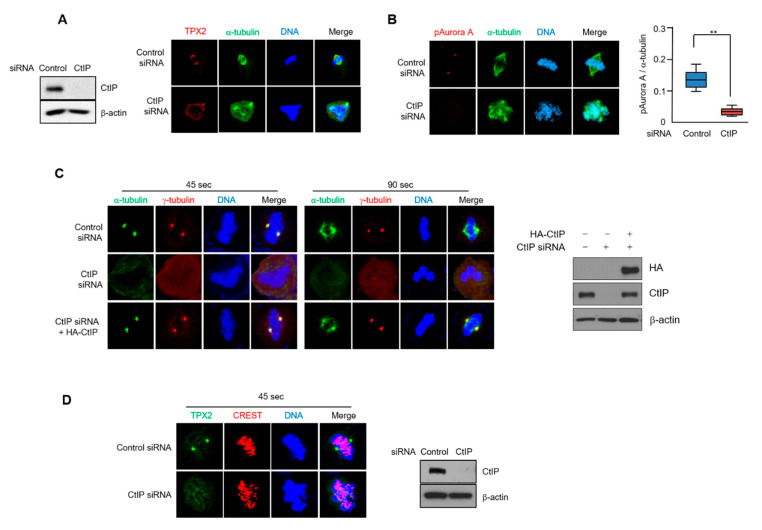
CtIP regulates MT dynamics. (**A**,**B**) Control and CtIP-depleted HeLa cells were fixed and stained with TPX2 and α-tubulin antibodies (**A**) or pAurora A and α-tubulin (**B**) antibodies. DNA was visualized using DAPI. Quantification of the fluorescence intensity of pAurora A normalized to the fluorescence intensity of α-tubulin. Graphs are represented the mean ± SD from three independents experiments. **, *p* < 0.01, compared to control cells. (**C**) Control HeLa cells, CtIP-depleted HeLa cells, and CtIP depleted HeLa cells reconstituted with HA-CtIP were incubated on ice for 1 h, rewarmed at 37 °C for 45 s and 90 s before fixation, and stained by immunofluorescence with α-tubulin (green) and γ-tubulin (red) antibodies. DNA was stained with DAPI. (**D**) Representative immunofluorescence images of control and CtIP depleted HeLa cells that were subject to 1 h of cold treatment on ice, rewarmed at 37 °C for 45 s before fixation, and stained by immunofluorescence with TPX2 (blue) and CREST (red) antibodies. DNA was stained with DAPI.

To characterize the biological relevance of the interaction between CtIP and TPX2, we examined the effects of CtIP depletion on the localization of TPX2 at the spindle poles. To this end, localization of TPX2 at the spindle poles was measured by fluorescence intensity using immunofluorescence microscopy. As shown in Figure 6A, cells depleted of CtIP showed that TPX2 is localized at the multiple poles and decreased in TPX2 localization at the spindle poles. Consequently, the localization of phosphorylated Aurora A, a key target protein of TPX2, was also markedly decreased at the spindle poles in the CtIP depleted cells (Figure 6B), suggesting that CtIP contributes to the efficient localization of TPX2 at the spindle poles. 

TPX2 is a downstream target of the small GTPase Ran, which releases TPX2 from the importin complex to assemble the mitotic spindles. The chromosomal protein RCC1 activates Ran and associates with chromatin to drive microtubule assembly [12]. Therefore, we examined the role of CtIP in Ran-dependent spindle assembly by depleting CtIP in HeLa cells and testing for RCC1 localization. As expected, RCC1 localized to chromatin during mitosis; however, there was no effect of CtIP depletion (Figure S3), which suggests that CtIP affects spindle assembly downstream of TPX2 signaling and is independent of the upstream regulator Ran.

TPX2 is a major regulator of spindle assembly [35]. The proper formation of bipolar spindles is critical for the accurate segregation of chromosomes into the two daughter cells during mitosis [14]. The spindles are maintained by both spindle assembly factors and MTs, and the MTs are assembled at both the centrosome and chromosomes. Because the centrosome is the primary microtubule organizing center, we looked for evidence that CtIP controls MT formation from the centrosome and kinetochore. Control and CtIP-depleted cells were chilled at 4 °C for 1 h to disrupt MTs and then rewarmed at 37 °C for 45 and 90 s to assess centrosome-based microtubule formation. In control cells, two centrosome-driven asters appeared when the cells were rewarmed at 37 °C for 90 s; however, the CtIP-or TPX-2-depleted cells showed very low MT intensity at the spindle poles (Figure 6C and Figure S4). The MT intensity at the spindle poles returned to normal when CtIP-depleted cells were complemented with wild-type CtIP. We also found that CtIP knockdown resulted in slow regrowth of microtubules when microtubules were allowed to regrow after warming at 37 °C for 2 and 5 min (Figure S5A,B). Likewise, as shown in Figure 6D, localization of TPX2 to the spindle was significantly reduced in the CtIP-depleted cells compared to that in the control cells, suggesting that CtIP is probably involved in maintaining proper spindle assembly by regulating TPX2 function.

## 4. Discussion

In this study, we identified a new role for CtIP in the regulation of microtubule dynamics at the centrosomal spindles. CtIP is a key DNA repair protein that promotes 5′ end resection, the initial step in HR. Recent studies have shown that CtIP, a binding partner of the MRN complex, is required for metaphase chromosome alignment in *Xenopus* egg extracts [31]. Our data from immunofluorescence microscopy data showed that CtIP colocalized with mitotic spindles throughout mitosis, and time-lapse microscopy showed a delayed metaphase-anaphase transition in CtIP-depleted HeLa cells, suggesting that CtIP plays an important role in proper mitotic progression.

Precise chromosome segregation in eukaryotic cells is important for maintaining chromosomal fidelity in daughter cells and is therefore protected by several highly coordinated mitotic processes [36]. When the cells enter mitosis, kinetochores assemble into centrosomes of duplicated chromatid pairs, and each kinetochore is captured by microtubule fibers from the opposite spindle poles. This linkage leads to chromosomal alignment at the spindle equator and subsequently to the onset of anaphase [37]. This highly regulated process has evolved with several factors to address and prevent and address erroneous chromosomal segregation, the failure of which is associated with tumorigenesis [38]. We observed coimmunoprecipitation of CtIP and TPX2 in vitro and colocalization at the mitotic spindle in vivo, indicating an important interaction between the two proteins during mitosis. This interaction was expected based on the results of quantitative BAC-GFP interactor (QUBIC) analysis, which showed that the interaction between CtIP and TPX2 was statistically significant [34,39]. When cells were depleted of CtIP, the localization of TPX2 at the spindle poles was reduced, suggesting that the delayed mitotic progression observed in CtIP-depleted cells may be due to a defect in the spindle assembly function of TPX2. 

TPX2 is a multifunctional protein essential for microtubule assembly during mitotic spindle formation [19,40], and it attracts other spindle components to the spindle [41]. TPX2 is relatively well-conserved and presents two main functional domains and several regulatory sequences [42,43]. The N-terminus is involved in binding and activating Aurora A [20,21,44,45]. TPX2-stimulated Aurora A activity is important for proper spindle assembly, centrosome function, and γ-tubulin ring complex activation [46,47,48]. The C-terminal half of TPX2 is responsible for binding to microtubules, stimulating augmin-mediated branching microtubule nucleation in *Xenopus laevis* egg extracts [49,50,51], whereas the terminal 35 amino acids interact with two mitotic kinesins Kif11/Eg5 and Kif15/Xklp2, mediating the proper spindle localization of these motors [18,26,52,53,54]. The Ran exchange factor RCC1 promotes the formation of RanGTP, which then causes TPX2 to dissociate from importins in the vicinity of chromosomes, thereby releasing its inhibitory effects [55,56,57]. This release enables TPX2 to interact with and activate Aurora A. Here, we showed that CtIP depletion impaired the localization of phospho-Aurora A to the spindle poles. However, CtIP depletion altered the localization of RCC1 to chromatin during mitosis. These results suggest that the function of CtIP in mitotic spindle assembly at the kinetochore plays a role in the TPX2/Aurora A signaling pathway after the binding of TPX2, rather than by increasing RanGTP concentrations to release TPX2 from importins. Furthermore, our results showed that CtIP-depleted cells exhibited mitotic defects such as chromosome misalignment, abnormal spindle morphology, anaphase bridges, aberration of the centrosome number, and defects in microtubule organization that are similar to the mitotic defects in TPX2 or Aurora A-depleted cells. The chromosomal misalignment phenotype observed in CtIP-deficient cells may be due to inhibitory microtubule formation as CtIP deficiency reduces microtubule regrowth. Although the mitotic defect caused by CtIP depletion arises from dysregulation of the TPX2-Aurora A signaling during mitosis, the possibility of other cellular mechanisms regulating mitotic spindle assembly by CtIP cannot be completely excluded. Taken together, in addition to the role of CtIP on maintaining genomic integrity by promoting HR, our findings reveal that CtIP mediates the proper execution of mitosis, which is also important for maintaining chromosome stability.

Although it is clear that TPX2 localizes to the nucleus during interphase and influences the chromatin environment with potential implications for DNA damage responses [35,58], the role of nuclear TPX2 remains largely unknown. Recently, it was reported that by suppressing p53 binding protein (53BP1), the TPX2-Aurora A heterodimer facilitates BRCA1-dependent DNA end resection, DNA damage-induced RAD51 focal nuclear accumulation, and HR [59]. In addition, the TPX2-Aurora A complex plays a role in stabilizing and protecting the stalled DNA replication forks that occur in response to DNA damage by negatively regulating 53BP1 function [59], indicating that TXP2 binds and counteracts 53BP1 to maintain HR and replication fork stability. Because TPX2 interacts with CtIP, and because, in contrast to 53BP1, CtIP promotes DNA end resection and replication fork stability, TPX2 may not only negatively regulate 53BP1 function but also positively regulate CtIP function. Accordingly, we are currently investigating whether the mechanism by which TPX2 regulates the DNA damage response includes the promotion of CtIP function. 

In summary, the results presented in this study have established CtIP as a novel and functional component in the regulation of microtubule dynamics during spindle assembly, thereby expanding the roles of CtIP to include the regulation of mitosis. Specifically, we showed that depletion of CtIP leads to delayed mitosis progression, multipolar spindles, and chromosome misalignment, as well as a defect in MT nucleation. Furthermore, we showed that CtIP binds and colocalizes with TPX2 during mitosis, contributing to the localization of TPX2 and its downstream target, Aurora A, at spindle poles. This work not only unveils a novel role for CtIP in maintaining microtubule dynamics but also highlights the significance of the importance of the role of the mitotic function of CtIP on genomic integrity. 

## Data Availability

Not applicable.

## Supplementary Materials

The following supporting information can be downloaded at: https://www.mdpi.com/article/10.3390/cells11182814/s1. Figure S1: CtIP contributes to the metaphase-anaphase transition. Figure S2: Depletion of CtIP increases the number of micronucleated cells. Figure S3: CtIP is not required for RCC1 recruitment to chromatin. Figure S4: TPX2 depletion decreases MT formation. Figure S5: CtIP regulates MT dynamics.
