# Supplementary material for: CtIP Regulates Mitotic Spindle Assembly by Modulating the TPX2-Aurora A Signaling Axis
Source: Cells. 2022 Sep 8;11(18):2814. doi: 10.3390/cells11182814 (PMC9497199; doi:10.3390/cells11182814)
Supplement: Supplementary file 1 [file cells-11-02814-s001.zip › cells-1814380-supplementary.pdf]

Supplementary Materials

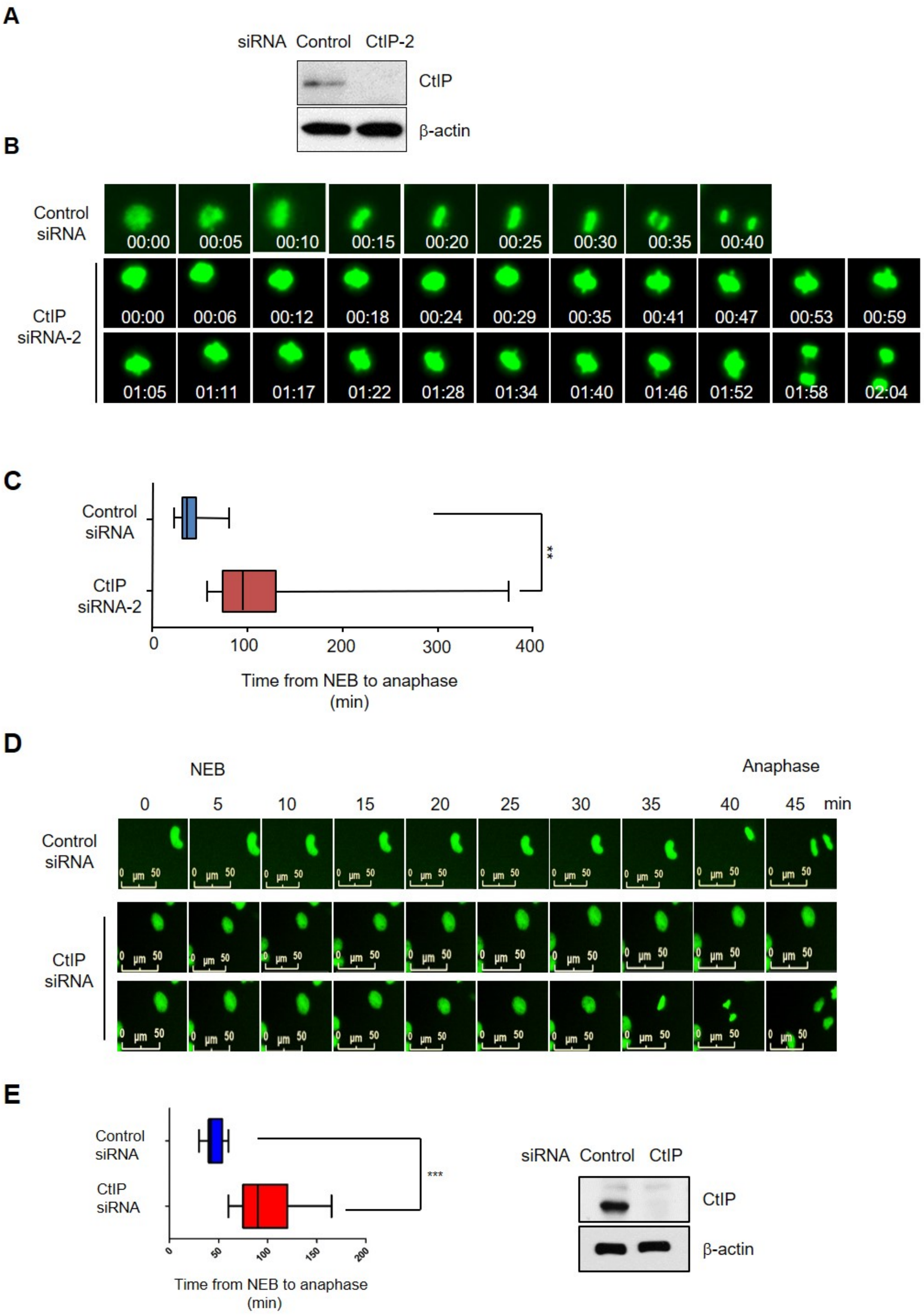

F

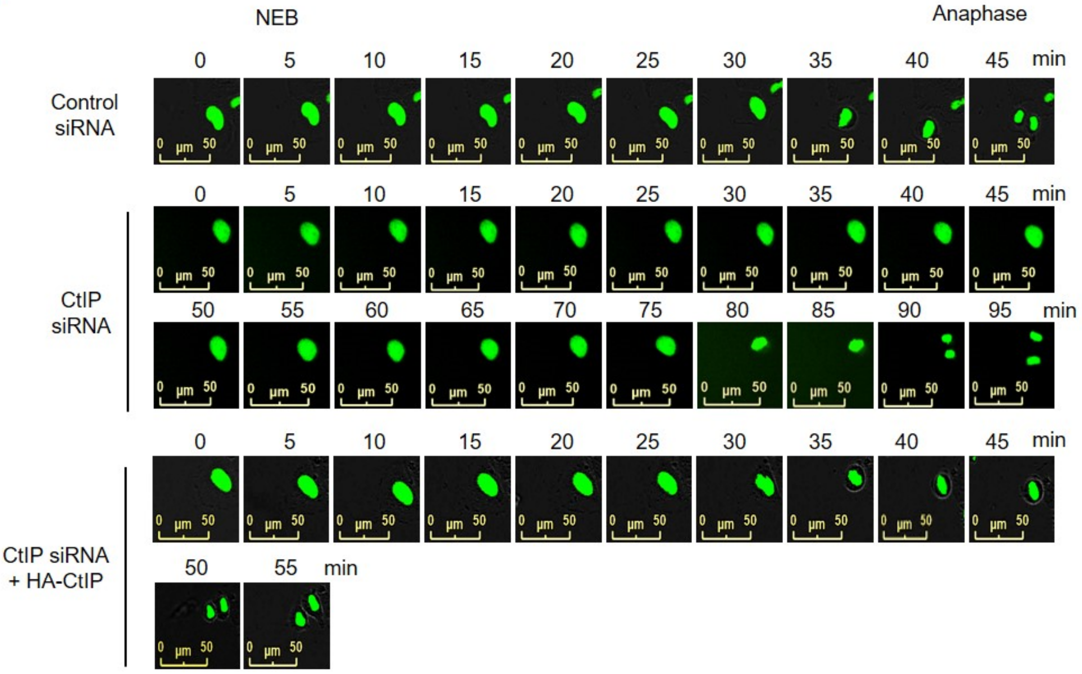

G

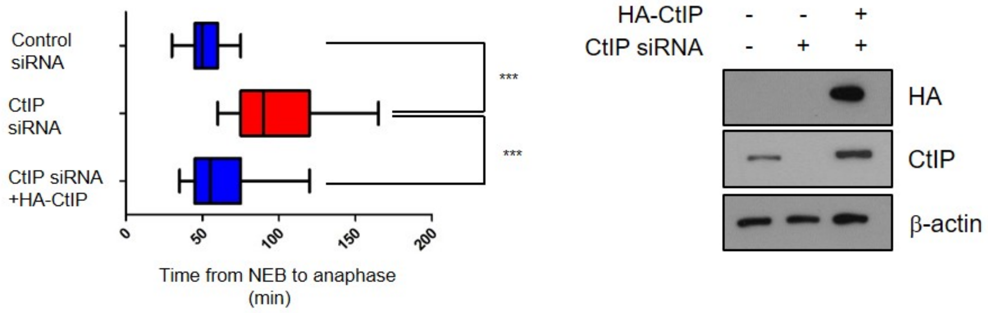

Supplementary Figure S1. CtIP contributes to the metaphase-anaphase transition. (A) Western blot analysis was performed to identify the CtIP expression levels in HeLa cells transfected with control siRNA or CtIP siRNA-2. **(B)** The progression of mitosis was monitored using time-lapse microscopy. HeLa cells transfected with control siRNA and CtIP siRNA-2 were grown in 12-well plates and transfected with GFP-tagged histone H2B. Fluorescent images were obtained every 5 min starting at the point of nuclear envelope breakdown. **(C)** A quantification of the time elapsed from nuclear envelope

breakdown to anaphase onset in HeLa cells transfected with control siRNA and CtIP siRNA-2. Graphs represent the mean ± SD from three independents experiments. ** $p <$ 0.01, compared to control cells. (**D)** Control U2OS cells and CtIP-depleted U2OS cells were analyzed for progression of mitosis as described in (**B**). **(E)** A quantification of the time elapsed from nuclear envelope breakdown to anaphase onset in control U2OS cells and CtIP-depleted U2OS cells. Graphs represent the mean ± SD from three independents experiments. *** $p < 0.001$, compared to control cells. (**F**) Control HeLa cells, CtIP-depleted HeLa cells, and CtIP depleted HeLa cells reconstituted with HA-CtIP were analyzed for progression of mitosis as described in (**B**). **(G)** A quantification of the time elapsed from nuclear envelope breakdown to anaphase onset in control HeLa cells, CtIP-depleted HeLa cells, and CtIP depleted HeLa cells reconstituted with HA-CtIP. Graphs represent the mean ± SD from three independents experiments. *** $p < 0.001$, compared to control cells.

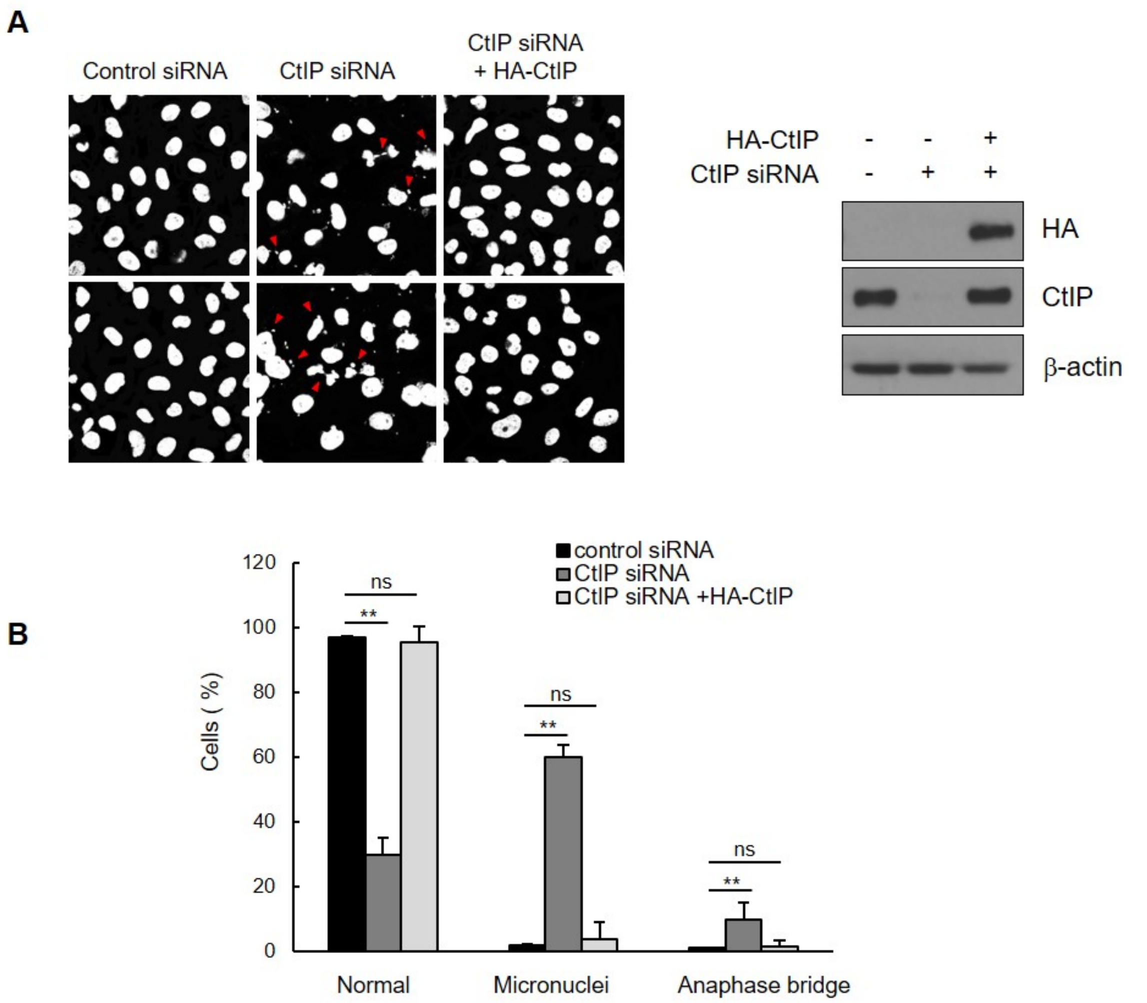

Supplementary Figure S2. Depletion of CtIP increases the number of micronucleated cells. (A) Images of mitotic defects in control HeLa cells, CtIP-depleted HeLa cells, and CtIP depleted HeLa cells reconstituted with HA-CtIP. Arrows indicate micronuclei and anaphase bridges. **(B)** Quantification of aberrant nuclear morphology in control and CtIP-depleted HeLa cells (n = at least 100 cells). $^{**}p < 0.01$.

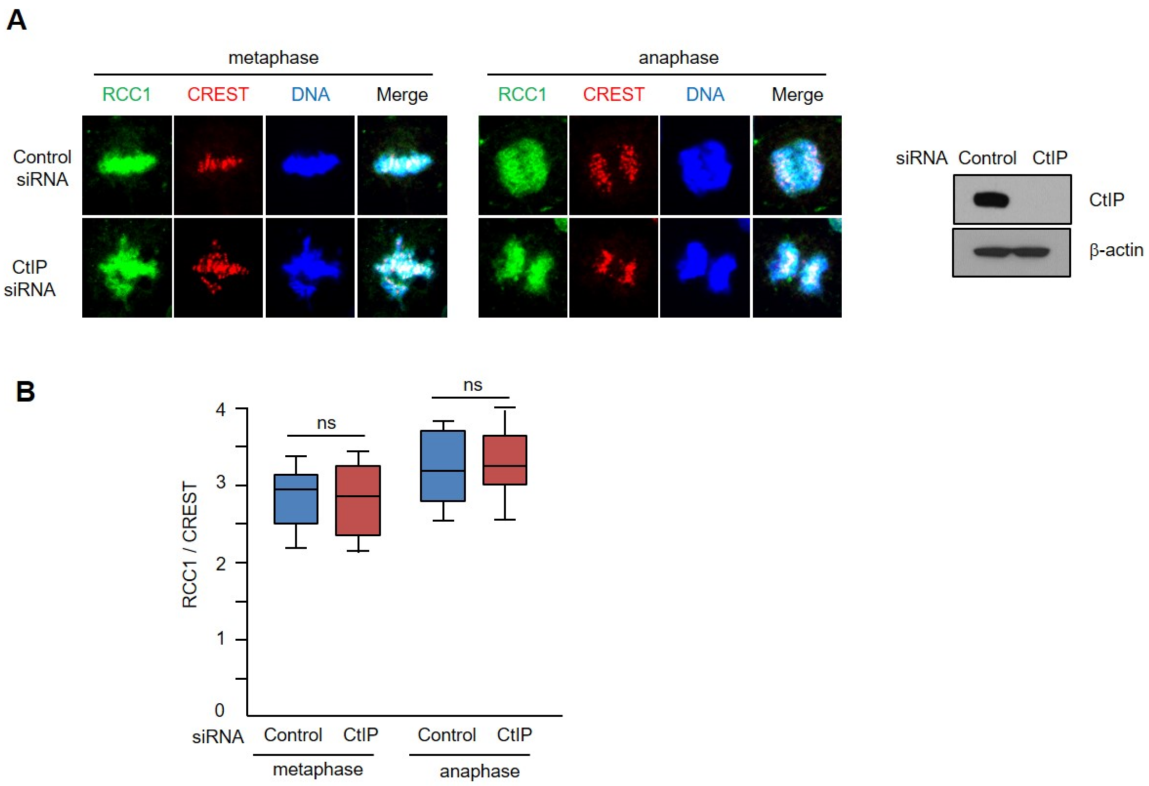

Supplementary Figure S3. CtIP is not required for RCC1 recruitment to chromatin. (**A**) Representative immunofluorescence images of control and CtIP depleted HeLa cells that were fixed and stained with RCC1 (green) and CREST (red) antibodies. DNA was visualized using DAPI. For confirm CtIP-depletion, western blot analysis was performed using anti-CtIP and anti-β-Actin antibodies. Western blot analysis was performed to measured the expression levels of CtIP in control and CtIP-depleted cells. (**B**) Quantification of the fluorescence intensity of RCC1 normalized to the fluorescence intensity of CREST. Graphs are represented the mean ± SD from three independents experiments. ns = nonsignificant.

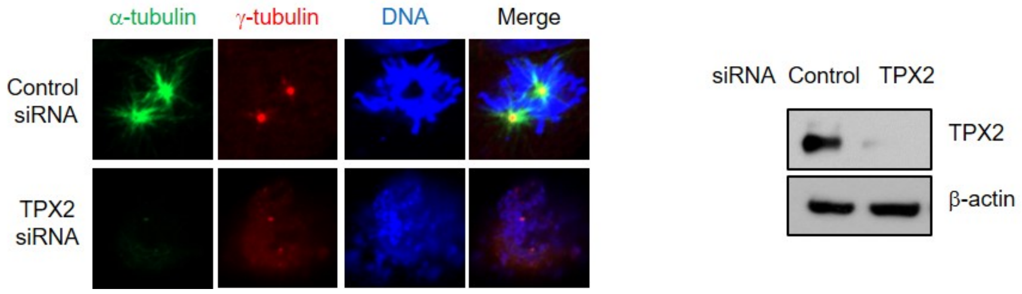

Supplementary Figure S4. TPX2 depletion decreases MT formation. Control and TPX2-depleted HeLa cells were incubated on ice for 1 h, rewarmed at 37 °C for 90 s before fixation, and stained by immunofluorescence with α-tubulin (green) and γ-tubulin (red) antibodies. DNA was stained with DAPI. Western blot analysis was performed to measured the expression levels of TPX2 in control and TPX2-depleted cells.

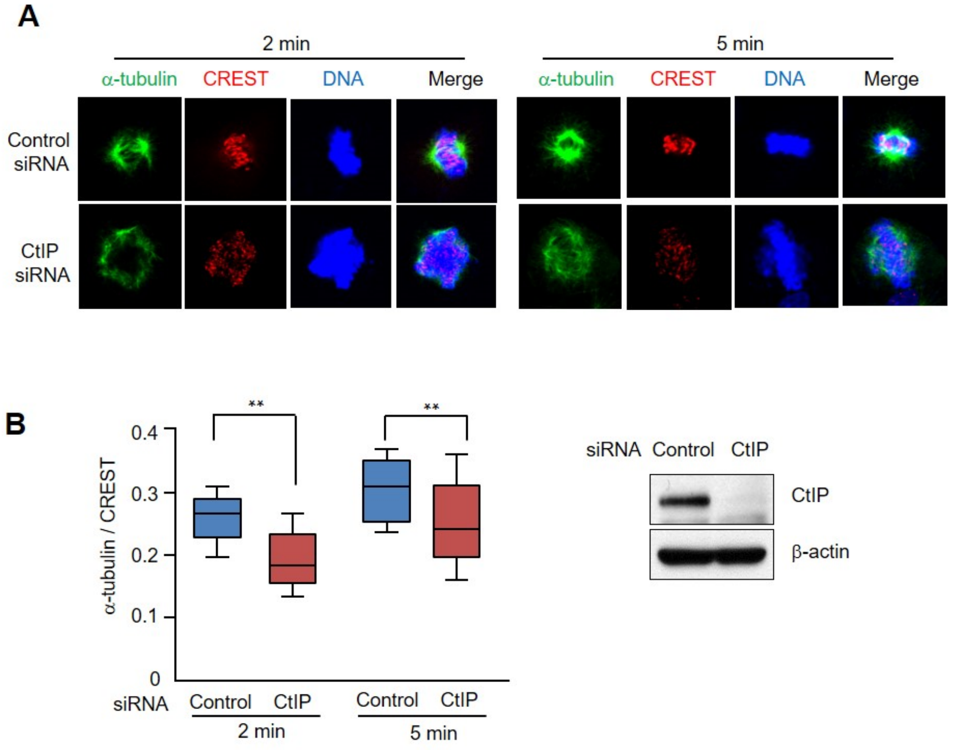

Supplementary Figure S5. CtIP regulates MT dynamics. (**A**) Control and CtIP-depleted HeLa cells were incubated on ice for 1 h, rewarmed at 37 °C for 2 min and 5 min before fixation, and stained by immunofluorescence with α-tubulin (green) and CREST (red)

antibodies. DNA was stained with DAPI. (**B**) Quantification of the fluorescence intensity of α-tubulin normalized to the fluorescence intensity of CREST. Graphs are represented the mean ± SD from three independents experiments. **p < 0.01.
